# Effects of artemisinin on ventricular arrhythmias in response to left ventricular afterload increase and microRNA expression profiles in Wistar rats

**DOI:** 10.7717/peerj.6110

**Published:** 2018-12-20

**Authors:** Xue Xu, Qiang Zhang, Huanqiu Song, Zhuo Ao, Xiang Li, Cheng Cheng, Maojing Shi, Fengying Fu, Chengtao Sun, Yuansheng Liu, Dong Han

**Affiliations:** 1 Department of Cardiology, Peking University People’s Hospital, Beijing, China; 2 National Center for Nanoscience and Technology, Beijing, China; 3 Emergency Department, Peking University People’s Hospital, Beijing, China; 4 Department of Physiology and Pathophysiology, Peking University School of Basic Medical Sciences, Beijing, China; 5 Department of Radiotherapy, Shandong Provincial Hospital Affiliated to Shandong University, Shandong University, Jinan, Shandong, China

**Keywords:** Artemisinin, Ventricular arrhythmia, Afterload increase, miRNA, Bioinformatics

## Abstract

**Background:**

Patients with dilated cardiomyopathy, increased ventricular volume, pressure overload or dysynergistic ventricular contraction and relaxation are susceptible to develop serious ventricular arrhythmias (VA). These phenomena are primarily based on a theory of mechanoelectric feedback, which reflects mechanical changes that produce alterations in electrical activity. However, very few systematic studies have provided evidence of the preventive effects of artemisinin (ART) on VA in response to left ventricle (LV) afterload increases. MicroRNAs (miRNAs) are endogenous small non-coding RNAs that regulate expression of multiple genes by suppressing mRNAs post-transcriptionally.

**Aims:**

The aims of this study were to investigate preventive effects of ART on mechanical VA and the underling molecular mechanisms of differentially expressed miRNAs (DEMs).

**Methods:**

For the study, 70 male Wistar rats were randomly divided into seven groups: group 1 was a control group (sham surgery); group 2 was a model group that underwent transverse aortic constriction (TAC) surgery; groups 3, 4, 5 and 6 were administered ART 75, 150, 300 and 600 mg/kg before TAC surgery, respectively; and group 7 was administered verapamil (VER) 1 mg/kg before TAC surgery. A ventricular arrhythmia score (VAS) was calculated to evaluate preventive effects of ART and VER on mechanical VA. The high throughput sequencing-based approach provided DEMs that were altered by ART pretreatment between group 2 and group 4. All predicted mRNAs of DEMs were enriched by gene ontology (GO) and Kyoto Encyclopedia annotation of Genes and Genomes (KEGG) databases. These DEMs were validated by a real time quantitative polymerase chain reaction (RT-qPCR).

**Results:**

The average VASs of groups 3, 4, 5, 6 and 7 were significantly reduced compared with those of group 2 (2.70 ± 0.48, 1.70 ± 0.95, 2.80 ± 0.79, 2.60 ± 0.97, 1.40 ± 0.52, vs 3.70 ± 0.67, *p* < 0.01, respectively). The three top GO terms were neuron projection, organ morphogenesis and protein domain specific binding. KEGG enrichment of the 16 DEMs revealed that MAPK, Wnt and Hippo signaling pathways were likely to play a substantial role in the preventive effects of ART on mechanical VA in response to LV afterload increases. All candidate DEMs with the exception of rno-miR-370-3p, rno-miR-6319, rno-miR-21-3p and rno-miR-204-5p showed high expression levels validated by RT-qPCR.

**Conclusions:**

Artemisinin could prevent mechanical VA in response to LV afterload increases. Validated DEMs could be biomarkers and therapeutic targets of ART regarding its prevention of VA induced by pressure overload. The KEGG pathway and GO annotation analyses of the target mRNAs could indicate the potential functions of candidate DEMs. These results will help to elucidate the functional and regulatory roles of candidate DEMs associated with antiarrhythmic effects of ART.

## Introduction

Ventricular arrhythmias (VA) include premature ventricular contraction (PVC), ventricular tachycardia (VT) and ventricular fibrillation (VF). VA are among the major causes of sudden cardiac death, which account for approximately half of cardiac mortalities (Zipes et al., 2006). Patients with dilated cardiomyopathy, increased ventricular volume, pressure overload (such as acute changes in blood pressure or transient aortic occlusion) or dysynergistic ventricular contraction and relaxation (such as myocardial infarction) are susceptible to develop serious VA (Kiseleva et al., 2000; Sarubbi et al., 1998; Sideris et al., 1987). These phenomena are primarily based on a theory of mechanoelectric feedback (MEF), which was defined as a process of mechanical changes affecting cardiac electrical activity. According to Laplace’s law, a rise in arterial blood pressure leads to a sudden increased afterload of the left ventricle (LV), which sequentially results in elevated LV systolic pressure (LVSP) and wall stress (Evans et al., 1995). Enhanced LV wall stress induces PVC, VT and VF within 10 s in isolated heart models using Langendorff technique (James & Jones, 1990; Salmon et al., 1997). As proved by previous studies, aortic occlusion increases LVSP and shortens action potential duration among in situ porcine (Horner et al., 1994) and human (Taggart et al., 1992) hearts.

Artemisinin (ART) is a classical antimalarial drug first isolated from *Artemisia annua* L by Chinese scientists in 1972 and initially used for malaria chemoprophylaxis and treatment. It was also used for many other therapeutic effects including antiviral, antiparasitic, antifungal, anti-inflammatory, anticancer and anti-immune activities (Ho et al., 2014; Tu, 2011; Wu et al., 2016). In the early stage, some investigators found that ART could significantly block Iks and Ikr ion channels of ventricular myocytes nonselectively and that these mechanisms play an important role in antiarrhythmic effects (Yang et al., 1998). However, very few systematic studies have provided evidence of the preventive effects of ART on VA in response to LV afterload increases.

MiRNAs are a group of single-stranded, highly conservative and small (∼22 nucleotides) non-coding RNAs, which serve as regulators of their target mRNAs by inhibiting the translation of mRNAs to functional protein or degrading target mRNAs (Zhang, Wang & Pan, 2007). Experimental research has demonstrated that miRNAs regulate cardiac excitability including conduction, repolarization, automaticity, Ca^2+^ handling, apoptosis and fibrosis by modulating target mRNAs expression (Kim, 2013; Latronico & Condorelli, 2010). In our study, we aimed to further investigate the potential molecular mechanisms of miRNAs involved in preventive effects of ART on MEF induced VA.

An animal model was established by us to evaluate the effects of ART and VER on PVCs and VTs in response to LV afterload increases by calculating average ventricular arrhythmia scores (VASs) of seven groups. In this study, we aimed to explore preventive effects of ART on mechanical VA in response to LV afterload increases and to determine differentially expressed miRNAs (DEMs) mediated signaling pathways and biological functions.

## Materials and Methods

### Animal groups

Overall, 70 male Wistar rats weighing between 225 and 275 g (average, 247.73 g) were obtained from Beijing Vital River Laboratory Animal Technology Co., Ltd (Beijing, China). The care of the animals complied with the principles of laboratory animal care formulated by the animal ethics committee of Peking University People’s Hospital {approval number: [2013] (65)}. Animals received water and food ad libitum until the research began, and they were maintained at a temperature of 24 °C in a 12 h light–dark cycle. A total of 70 Wistar rats were randomly assigned as follows (10 rats/group):

Group 1: (control group) sham surgery, group 2: (model group) transverse aortic constriction (TAC) surgery, group 3: ART 75 mg/kg+TAC surgery, group 4: ART 150 mg/kg+TAC surgery, group 5: ART 300 mg/kg+TAC surgery, group 6: ART 600 mg/kg+TAC surgery, group 7: VER 1 mg/kg+TAC surgery. The ART-pretreated rats were administered by oral gavage once daily for 3 days. The rats of the VER-pretreated groups were slowly administered the medication via tail vein injections over a 10 min period before TAC surgery. The dosage of VER was calculated in accordance with methods reported previously (Bhatti & Foster, 1997; Deng et al., 2017).

### Drugs and chemicals

Artemisinin was purchased from Tokyo Chemical Industry Co., Ltd (Tokyo, Japan) and VER was purchased from Shanghai Harvest Pharmaceutical Co., Ltd (Shanghai, China). The VER was dissolved in 0.9% saline solution (Beijing Leagene Biotechnology Co., Ltd, Beijing, China) and ART in Dimethyl Sulfoxide, separately. Heparin and chloral hydrate were obtained respectively from Beijing Solarbio Science & Technology Co., Ltd (Beijing, China) and Sinopharm Chemical Reagent Co., Ltd (Shanghai, China). TRIzol reagent and RNAlater were purchased from Thermo Fisher Scientific Inc (Waltham, MA, USA). Fresh drug solutions were prepared at the beginning of each experiment during the entire experimental period.

### TAC surgical procedure

The rats were anesthetized with chloral hydrate (10%), which was administered intraperitoneally at a dose of 3 ml/kg. By clamping the subcutaneous needles with electrodes, a standard electrocardiograph (ECG) lead (Lead II) between the left hind leg and the right foreleg was continuously recorded throughout the surgery. ECG was used to assess the susceptibility to VA related to mechanoelectrical feedback and the side effects of ART and VER. We placed a polyethylene-50 (PE-50) catheter into the right common carotid artery and inserted it into the LV cavity through the aortic valve to measure LV pressure. The PE-50 catheter was filled with 0.3% heparin saline (3 ml/kg), connected to a transducer and, in turn, to a data acquisition and preservation system (Powerlab; ADInstruments Pty Ltd, Bella Vista, NSW, Australia) via a three-way tube. Subsequently, we elicited a rapid pressure overload-induced ventricular arrhythmia using an aortic banding procedure. A mid-sternal thoracotomy was executed to expose the ascending aorta for TAC surgery. The aortic arch between the origin of the left common carotid and right brachiocephalic artery was freed by vascular forceps and threaded by a silk suture (Deknatel 2-0). A 0.7 mm-diameter wire was placed parallel to the aortic arch. A silk suture was tied against the wire and the aortic arch; the wire was then promptly pulled out to constrict the aortic arch. The silk suture was loosened after 5 min of constriction. ECG parameters were recorded, digitalized and analyzed by Labchart data analysis software version 7.2 (ADInstruments Pty Ltd, Bella Vista, NSW, Australia). The sham-surgery group underwent the same surgical process except tightening of the suture was performed first (Fischer et al., 1997). The surgical incisions were closed by suturing, and they were wiped with a povidone-iodine solution. Benzylpenicillin (100,000 U/kg) was administered intramuscularly for 3 days to the rats in all groups. Two days later, all rats were anesthetized, and the hearts were isolated from the body. The LV apex tissue was dissected and placed into centrifuge tubes with RNAlater solutions for RNA stabilization and stored at 4 °C overnight. Then tissues were stored at −80 °C for long-term storage.

### Definitions and measurements and of the ECG characteristics

PR intervals and heart rate corrected QT (QTc) intervals were calculated in all groups before TAC surgery. PR interval is the period that extends from the beginning of the P wave until the QRS complex initiation. The QT interval is a time scale between the start of the Q wave and the end of the T wave. The QTc interval was calculated by applying Bazett’s formula, namely, QTc = QT/RR^1/2^ (Bealer & Little, 2013).

Walker et al. (Curtis et al., 2013) published the Lambeth conventions to define VA in animal models. PVC is defined as a QRS wave that is different in shape from the normal ventricular complex and is premature in relation to the preceding ventricular complex. VT is defined as a run of four or more consecutive PVCs in animals. A VA scoring system modified by Yang et al. (1995) was used to execute a comparative quantitative analysis of VASs and was defined as follows: 0, no arrhythmias; 1, occasional VPC; 2, frequent VPCs (three or more VPCs within 1 min); 3, VT (one or two episodes); 4, VT (three to five episodes); 5, VT (more than five episodes). The most severe type of ventricular arrhythmia observed in each rat was assigned as one corresponding score during the entire TAC surgery period (5 min). The VASs and the number of rats that exhibited different types of atrioventricular blocks (AVBs) were determined in all groups.

### RNA extraction and miRNA library sequencing

Three LV tissues were randomly selected in groups 2 and 4 (indicated by the results in “Effects of ART and VER on VASs,” mean VAS in group 4 was the lowest in ART pretreated groups). Total RNA was isolated from the LV apex using TRIzol Reagent according to the manufacturer’s protocol. RNA purity was estimated by ND-1000 Nanodrop. RNA concentration and integrity were evaluated by Agilent 2200 TapeStation (Agilent Technologies, Santa Clara, CA, USA). Qualified RNAs were connected to a 3′-adapter and followed by a 5′-adapter closely. Subsequently, the adapter-ligated RNAs were reverse-transcribed to cDNAs and cDNAs were amplified with a low-cycle using a reverse transcription-polymerase chain reaction. Then the cDNA products with a length of 18–40 nt were selected by polyacrylamide gel electrophoresis followed by the introduction of a NEBNext® Multiplex Small RNA Library Prep Set for Illumina® (Illumina, San Diego, CA, USA). We constructed and evaluated a cDNA library using the Agilent 2200 TapeStation. The cDNA library was diluted to 10 pM for sequencing (1 × 50 bp) on a HiSeq 2500 platform.

### Analysis of sequencing data and DEMs

All the raw reads were obtained from the sequencing machine. After removing adaptors at both ends of the sequences, eliminating low quality reads and cleaning up the pollutant, we obtained clean reads. We aligned the clean reads to the miRBase version 21.0 database to annotate the known miRNAs and calculate the expressions of miRNAs. A bioconductor tool referred to as empirical analysis of digital gene expression in R (Robinson, McCarthy & Smyth, 2010) was performed on normalized expression profiles to identify DEMs between the samples of group 4 and group 2. Significant differences in miRNAs expression were carefully filtered using a normalized fold change value which was named as |[log2(fold change)]| = |log 2 (mean expression ratio (group 4/group 2))| > 1 and *p* < 0.05.

### Target genes prediction, functional and pathway enrichment analysis

We used the TargetScan, miRDB, miRTarBase and miRWalk algorithms to predict target mRNAs of DEMs. The intersections of target genes predicted by the above four algorithms were selected to be the candidate mRNAs. To further understand the signaling pathways and biological functions enriched from the candidate mRNAs, GO and Kyoto Encyclopedia annotation of Genes and Genomes (KEGG) enrichment analyses were carried out using the DAVID gene annotation tool.

### Construction of miRNA-mRNA integrated network

A MiRNA-mRNA interaction network was generated. The network consisted of 16 miRNAs manifested by their names, 1,846 pink nodes for mRNAs and each line represented the relationship between each miRNA and mRNA.

### DEMs validated by RT-qPCR

Real time quantitative polymerase chain reaction (RT-qPCR) was used to quantify the expression of 16 DEMs in three tissues of group 2 and group 4. Following this, one μg of total RNA extracted from each sample was reverse-transcribed using a miDETECT A Track™Uni-RT Primer. The reactions were incubated at 42 °C for 60 min and at 72 °C for 10 min. Real-time PCR was performed in triplicate wells using CFX96 (Bio-Rad Laboratories, Hercules, CA, USA) according to the protocol. In a 10 μl reaction mixture, 1.0 μl of cDNA was used as a template, with five μl of SYBR green Supermix, 0.4 μl of specific forward primer, 0.4 μl of reverse primer and treated utilizing the following procedure: 95 °C for 10 min, followed by 40 cycles of 95 °C for 10 s, 60 °C for 20 s and 70 °C for 10 s. The 2^−ΔΔCt^ method was carried out to calculate the relative expressions of DEMs (vs U6 snRNA).

### Statistical methods

Electrocardiograph characteristics and LV pressure in all groups were analyzed by one-way ANOVA analyses. We used a two-sided Fisher’s exact test to select the significant pathways and classify functional categories. DEMs expression of groups 2 and four validated by RT-qPCR were analyzed by a Student’s *t*-test. All results were presented as means and standard deviations. A value of *p* < 0.05 was considered significantly different.

## Results

### Characteristics of ECG changes during TAC surgery

**Figure 1:**
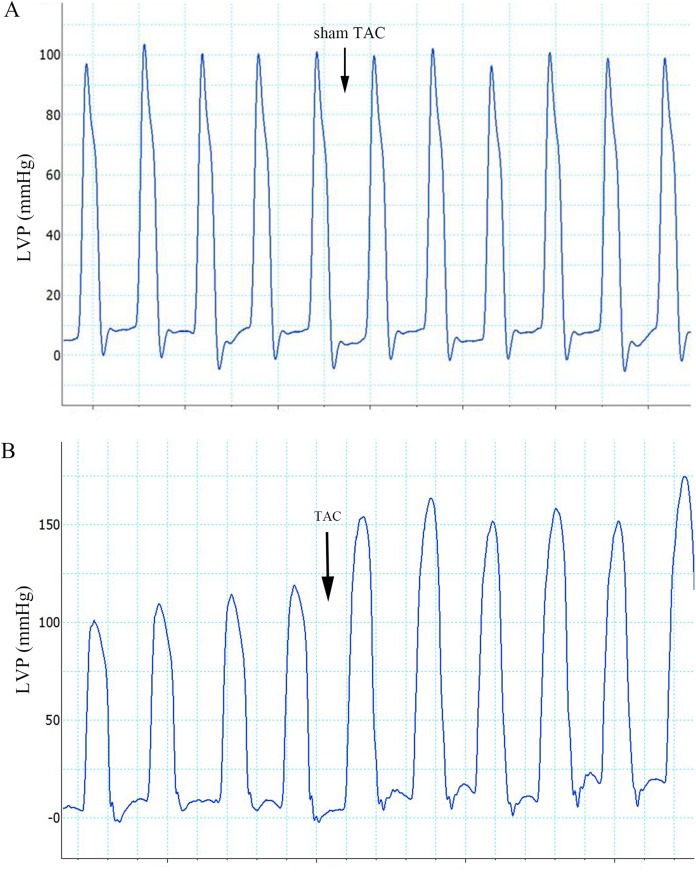
Effects of sham TAC surgery and TAC surgery on LVPs. (A) LVP in rats of group 1 after sham TAC surgery did not show obvious changes. The onset of sham TAC surgery is noted by the arrow. (B) Elevated LVP in response to LV afterload increases (induced by TAC surgery) in rats of group 2. The onset of TAC surgery is noted by an arrow. LVP, left ventricular pressure; TAC, transverse aortic constriction.

**Figure 2:**
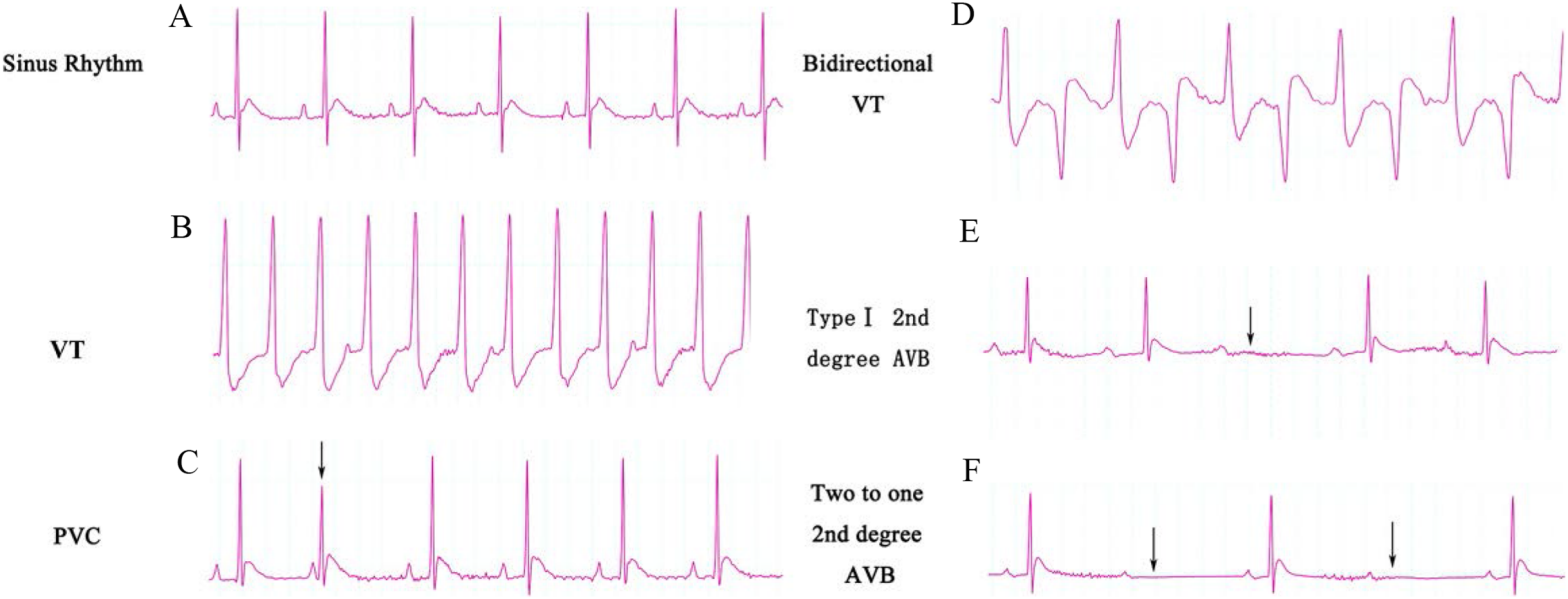
ECG categories that appeared in this experiment. (A) Sinus rhythm; (B) VT, ventricular tachycardia; (C) PVC, premature ventricular contraction; (D) Bidirectional VT; (E) Type I second degree AVB; (F) two to one second degree AVB. AVB, atrioventricular block.

The ECG graphs were used to identify where a sinus rhythm appeared in all rats before TAC surgery. LV pressure increased rapidly during TAC surgery (Fig. 1). VA occurred in response to increased afterload as indicated by PVCs (premature QRS complex noted by arrow in Fig. 2C) and VTs (Figs. 2B and 2D); however, AVBs were not generated. Type I second degree AVBs (absent QRS complex noted by arrow in Fig. 2E) and two to one second degree AVBs (absent QRS complex noted by arrow in Fig. 2F) were both observed in the ART and VER pretreated groups (Fig. 2).

### Effects of ART and VER on PR and QTc intervals

**Figure 3:**
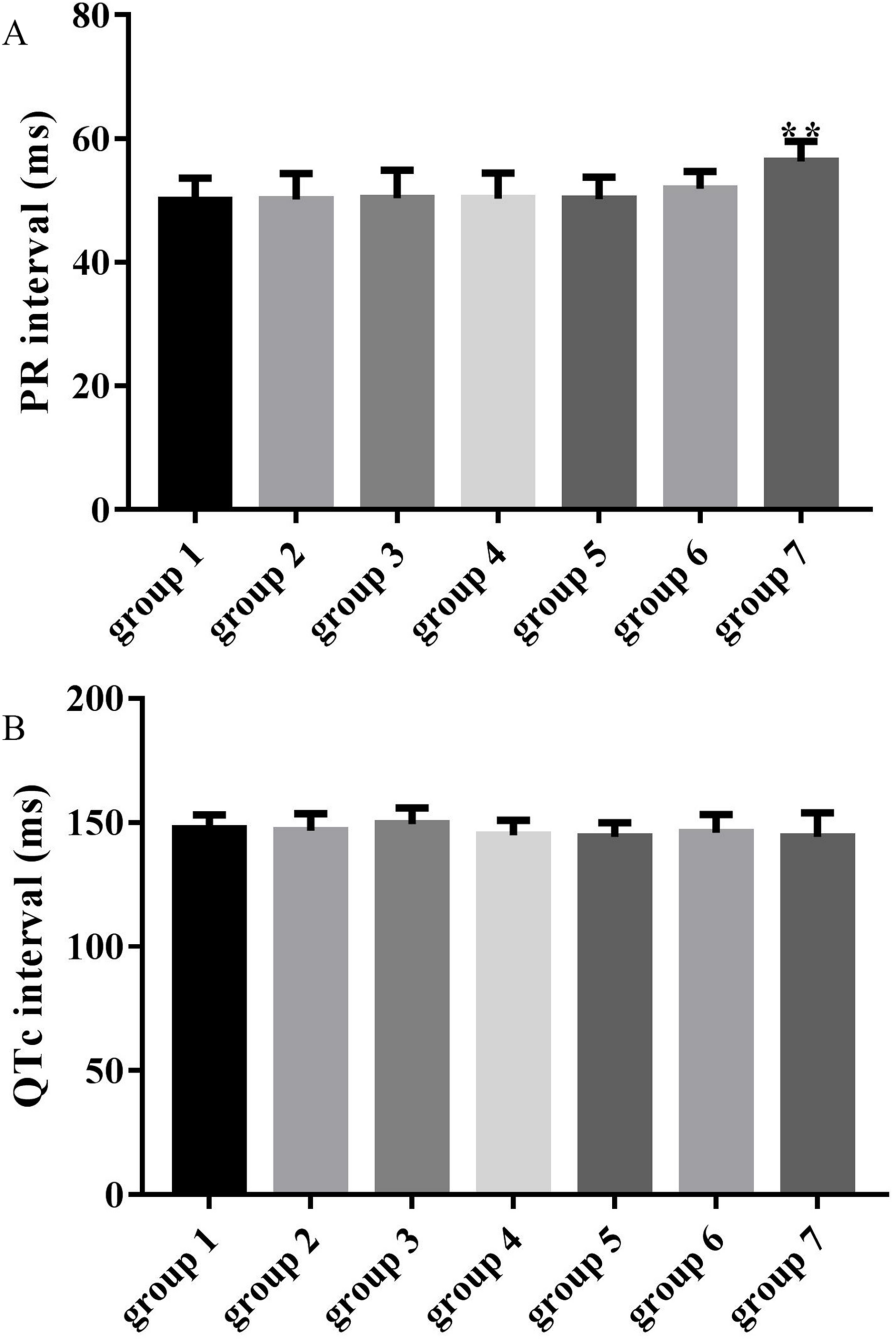
Effects of ART and VER on PR and QTc intervals before TAC surgery. (A) PR intervals in seven groups pretreated with saline or drugs before TAC surgery. (B) QTc intervals in seven groups pretreated with saline or drugs before TAC surgery. **p < 0.01 vs group 1. TAC, transverse aortic constriction.

The average PR intervals in groups 3, 4, 5 and 6 were unchanged compared with the control group (50.37 ± 4.54, 50.30 ± 4.13, 50.27 ± 3.47, 51.89 ± 2.80 vs 50.00 ± 3.66, *p* > 0.05), while the PR interval was significantly prolonged in group 7 compared with the control group (56.31 ± 3.28 vs 50.00 ± 3.66, *p* < 0.01) as shown in Fig. 3A. QTc intervals in groups 3, 4, 5, 6 and 7 were unchanged compared with the control group (149.34 ± 6.61, 144.88 ± 6.07, 144.28 ± 5.57, 145.95 ± 7.30 and 144.17 ± 9.79 vs 147.39 ± 5.62, *p* > 0.05) as shown in Fig. 3B.

### Preventive effects of ART and VER on ventricular arrhythmias

**Figure 4:**
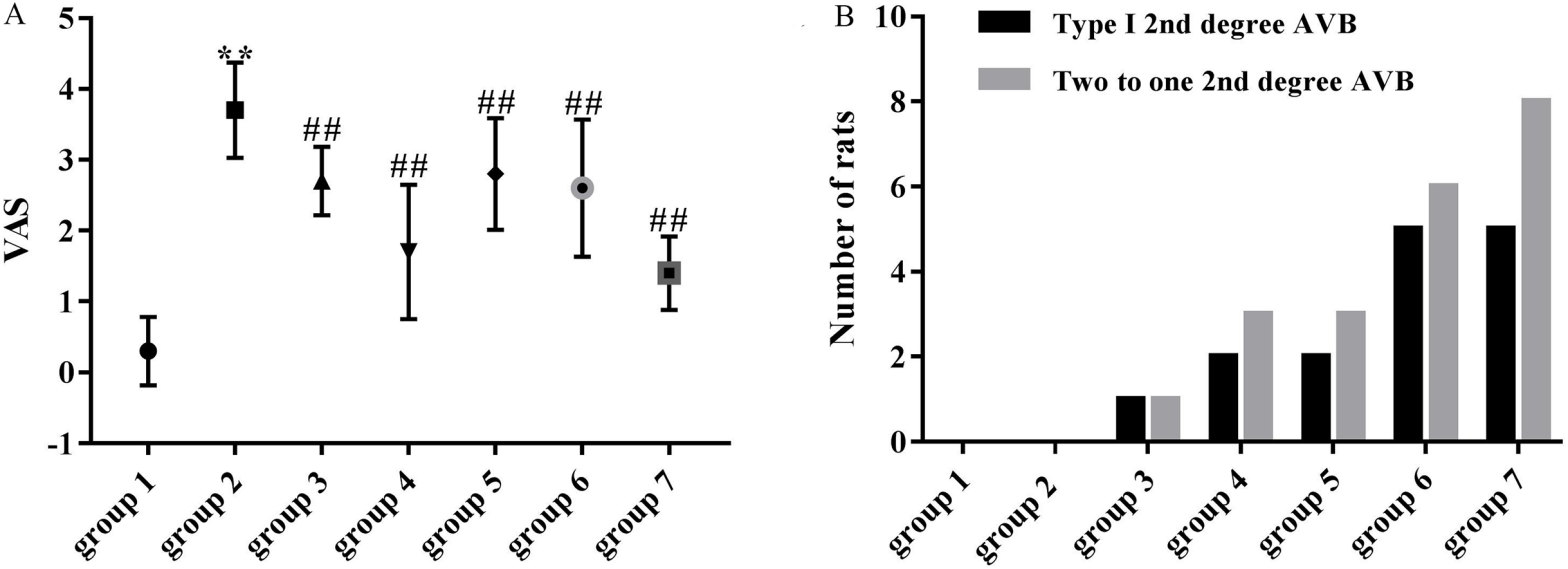
Effects of ART and VER on VAS and number of rats that presented with type I second degree AVB and two to one second degree AVB in all groups. (A) Obvious effects of ART (75, 150, 300 and 600 mg/kg) and VER (1 mg/kg) on VAS in response to LV afterload increases. (B) Number of rats that presented with type I second degree AVB and two to one second degree AVB in each group. **p < 0.01 vs group 1, ^##^*p* < 0.01 vs group 2. VAS, ventricular arrhythmia score; AVB, atrioventricular block.

As shown in Fig. 4A, the mean VAS was significantly higher in the model group (3.70 ± 0.67) compared with the control group (0.30 ± 0.48). VASs in group 3 (2.70 ± 0.48 vs 3.70 ± 0.67, *p* < 0.01), group 4 (1.70 ± 0.95 vs 3.70 ± 0.67, *p* < 0.01), group 5 (2.80 ± 0.79 vs 3.70 ± 0.67, *p* < 0.01), group 6 (2.60 ± 0.97 vs 3.70 ± 0.67, *p* < 0.01) and group 7 (1.40 ± 0.52 vs 3.70 ± 0.67, *p* < 0.01) were significantly decreased compared with the model group (3.70 ± 0.67).

### Number of rats that presented with AVBs

Third-degree AVBs were not observed in ART and VER pretreated groups. 1/10 rats in group 3, 2/10 rats in group 4, 2/10 rats in group 5, 5/10 rats in group 6 and 5/10 rats in group 7 presented with type I second degree AVB. In addition, 1/10 rats in group 3, 3/10 rats in group 4, 3/10 rats in group 5, 6/10 rats in group 6, 8/10 rats in group 7 presented with two to one second degree AVB (Figs. 3E and 3F). ART pretreatment with a dosage of 150 mg/kg (group 4) appeared to have the best preventive effect as the average numbers of rats that presented with type I second degree AVB and two to one second degree AVB were the lowest (Fig. 4B).

### Deep sequencing results of miRNAs

**Figure 5:**
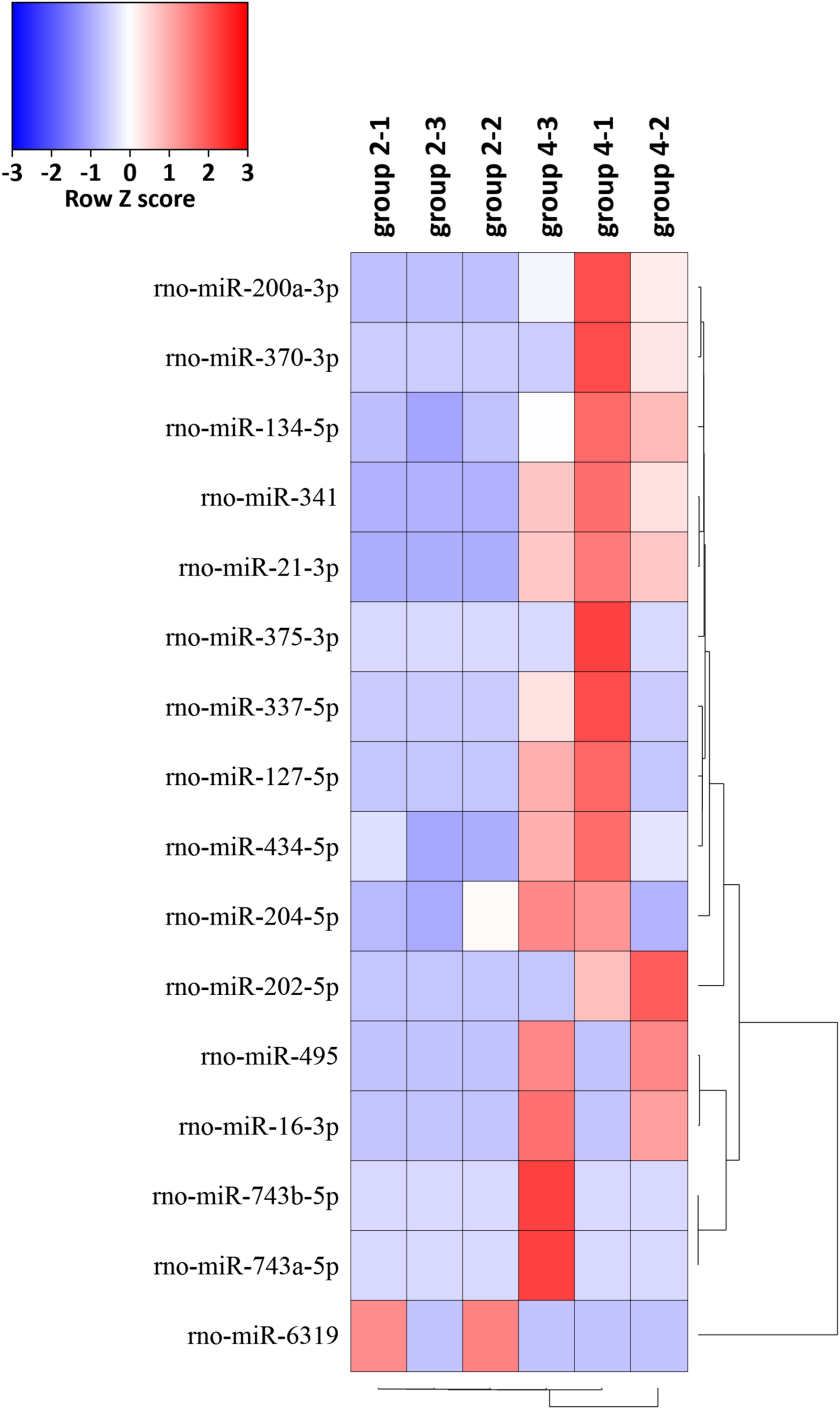
Hierarchical cluster analysis of 16 DEMs related to the preventive effects of ART on mechanical VA in response to LV afterload increases based on expressions of six tissue samples in groups 2 and 4. Red represents increased miRNA expression and blue represents reduced miRNA expression. MiRNAs limited to |log2(foldchange)| > 1 and *p* < 0.05 were displayed in the heat map. DEMs, differentially expressed miRNAs.

The expression levels of the 16 miRNAs [|log2(foldchange)| > 1 and *p*-value < 0.05] in six samples were shown by the heat map (Fig. 5). We identified 16 DEMs in 765 known miRNAs, which were changed significantly [|log2(foldchange)| > 1, *p* < 0.05)] between group 2 and group 4. Expression levels of rno-miR-200a-3p, rno-miR-341, rno-miR-21-3p, rno-miR-202-5p, rno-miR-370-3p, rno-miR-337-5p, rno-miR-127-5p, rno-miR-495, rno-miR-16-3p, rno-miR-204-5p, rno-miR-743b-5p, rno-miR-134-5p, rno-miR-434-5p, rno-miR-743a-5p and rno-miR-375-3p were significantly upregulated and rno-miR-6319 was downregulated in group 4 compared with group 2 (Table 1).

**Table 1:** Sixteen significantly differentially expressed miRNAs between group 2 and group 4.

miRNA_ID	Up/down	Log2(foldchange)	*p*-Value
rno-miR-200a-3p	Up	7.4174	3.80E-05
rno-miR-341	Up	6.9806	7.05E-05
rno-miR-21-3p	Up	6.4468	0.000449764
rno-miR-202-5p	Up	7.5349	0.001903303
rno-miR-370-3p	Up	6.6244	0.010916208
rno-miR-337-5p	Up	6.541	0.012188281
rno-miR-127-5p	Up	6.4321	0.012733603
rno-miR-495	Up	5.8694	0.016790488
rno-miR-16-3p	Up	5.8076	0.018679521
rno-miR-204-5p	Up	1.1891	0.020165031
rno-miR-743b-5p	Up	7.166	0.022456989
rno-miR-6319	Down	−5.7876	0.02430747
rno-miR-134-5p	Up	1.4378	0.035235138
rno-miR-434-5p	Up	1.4075	0.03707316
rno-miR-743a-5p	Up	6.6054	0.0412662
rno-miR-375-3p	Up	6.8176	0.046167278

### Prediction of target genes

These results showed that 16 DEMs had 1,846 target genes.

### Functional bioinformatics analysis

**Figure 6:**
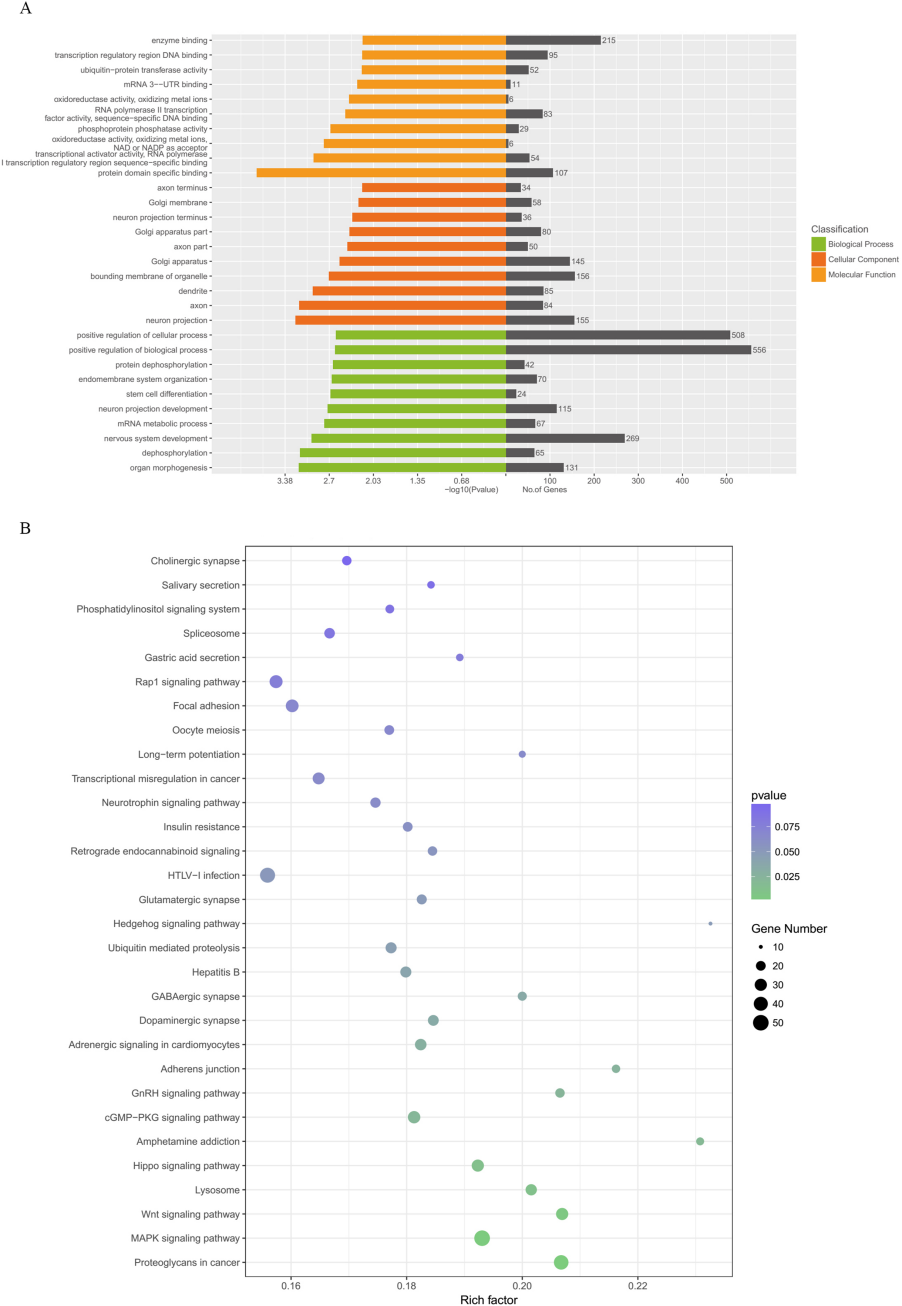
GO categories and KEGG analysis of DEMs. (A) Biological processes, cellular components and molecular functions of the candidate target genes which were enriched by GO categories. (B) Signaling pathways enriched from candidate target genes using KEGG analysis. GO, gene ontology; KEGG, Kyoto Encyclopedia annotation of Genes and Genomes; miRNA, microRNA. DEMs, differentially expressed miRNAs.

Figure 5 illustrates the results of the GO annotation analysis; 1,846 predicted target genes were significantly enriched in terms of 361 biological processes, 61 cellular components and 62 molecular functions. Notably, organ morphogenesis, dephosphorylation, neuron projection, axon, protein domain specific binding and transcriptional activator activity and RNA polymerase II transcription regulatory region sequence-specific binding seemed to be the most significantly enriched with GO terms (Fig. 6A). Among the 1,846 predicted target genes, according to the KEGG enrichment results, the main enriched pathways were proteoglycans in cancer, MAPK signaling pathway, the Wnt signaling pathway, lysosome and the Hippo signaling pathway (Fig. 6B; Table 2).

**Table 2:** Enriched KEGG pathway analysis of the predicted target genes of 16 differentially expressed miRNAs.

Pathway name	Gene number	*p*-Value
Proteoglycans in cancer	43	0.00103411
MAPK signaling pathway	50	0.001562279
Wnt signaling pathway	30	0.005403972
Lysosome	26	0.011992977
Hippo signaling pathway	30	0.012670714
Amphetamine addiction	15	0.019603748
Axon guidance	24	0.312966213
cGMP-PKG signaling pathway	31	0.022057892
GnRH signaling pathway	19	0.023634904
Adherens junction	16	0.026023104
Adrenergic signaling in cardiomyocytes	27	0.029128596
Dopaminergic synapse	24	0.034215625
GABAergic synapse	18	0.03436474
Hepatitis B	25	0.039361553
Ubiquitin mediated proteolysis	25	0.044555283
Hedgehog signaling pathway	10	0.048989119
Glutamatergic synapse	21	0.049250163

**Figure 7:**
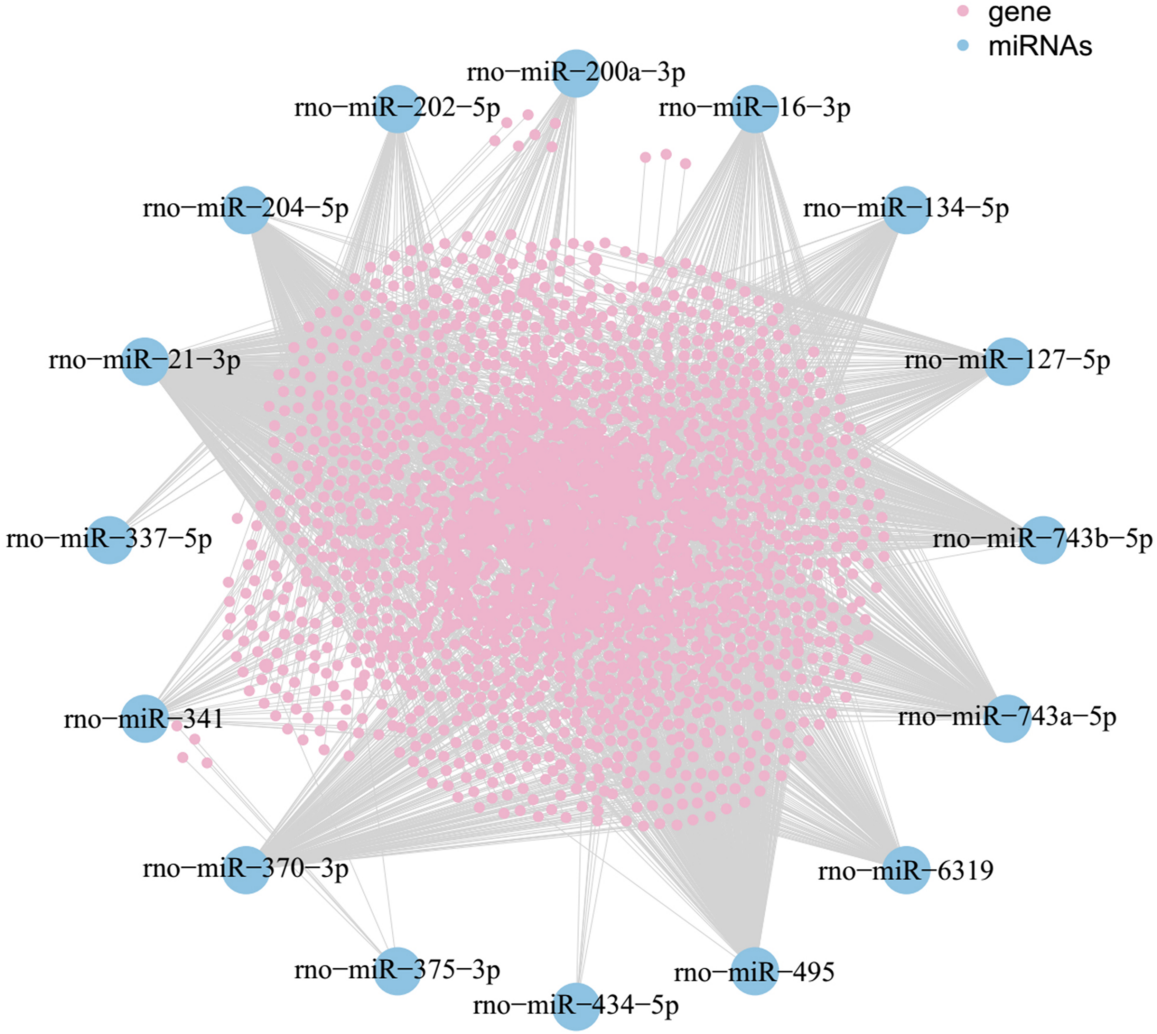
MiRNA-mRNA network. Blue nodes represent miRNAs, while pink nodes represent mRNAs. The more connections between miRNAs and genes, the more links within the network.

**Figure 8:**
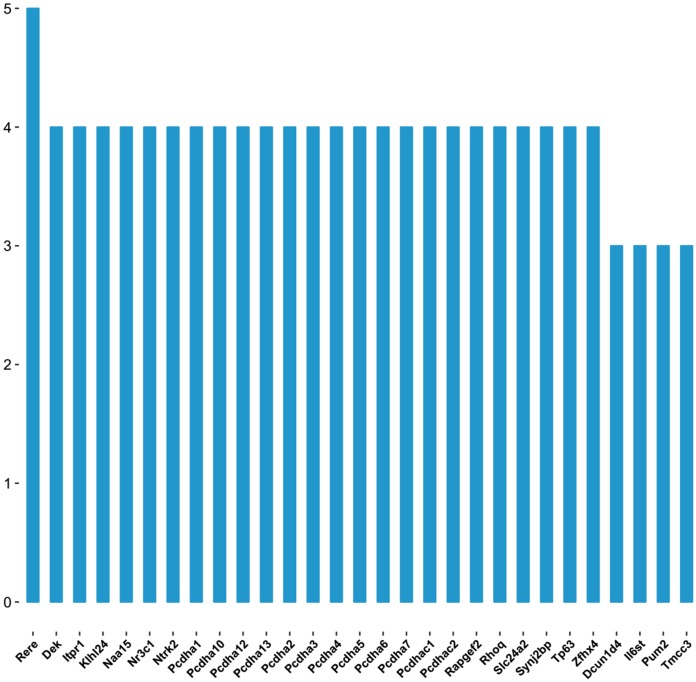
The number of genes linked to target genes. The vertical axis represents number of genes linked to the target genes, and horizontal axis represents the name of target genes.

As shown in the miRNA-mRNA regulatory network, rno-miR-495 regulates most of the 431 target genes (Fig. 7). Among candidate target genes in the present study, 25/1,846 genes have four or more miRNA predictive binding sites; Rere in particular can be one of the target genes in up to 5/16 miRNAs, and 24 genes have 4/16 miRNA binding sites (Fig. 8; Table 3).

**Table 3:** Target genes that are controlled and regulated by more than four microRNAs.

Target genes	miRNA counts	miRNA
Rere	5	rno-miR-200a-3p, rno-miR-202-5p, rno-miR-204-5p, rno-miR-370-3p, rno-miR-495
Pcdha5	4	rno-miR-127-5p, rno-miR-21-3p, rno-miR-495, rno-miR-743a-5p
Ntrk2	4	rno-miR-204-5p, rno-miR-21-3p, rno-miR-495, rno-miR-6319
Pcdha6	4	rno-miR-127-5p, rno-miR-21-3p, rno-miR-495, rno-miR-743a-5p
Zfhx4	4	rno-miR-202-5p, rno-miR-204-5p, rno-miR-495, rno-miR-743b-5p
Pcdha4	4	rno-miR-127-5p, rno-miR-21-3p, rno-miR-495, rno-miR-743a-5p
Pcdha3	4	rno-miR-127-5p, rno-miR-21-3p, rno-miR-495, rno-miR-743a-5p
Pcdha7	4	rno-miR-127-5p, rno-miR-21-3p, rno-miR-495, rno-miR-743a-5p
Tp63	4	rno-miR-204-5p, rno-miR-21-3p, rno-miR-6319, rno-miR-743a-5p
Pcdha1	4	rno-miR-127-5p, rno-miR-21-3p, rno-miR-495, rno-miR-743a-5p
Pcdha13	4	rno-miR-127-5p, rno-miR-21-3p, rno-miR-495, rno-miR-743a-5p
Dek	4	rno-miR-127-5p, rno-miR-341, rno-miR-6319, rno-miR-743a-5p
Slc24a2	4	rno-miR-134-5p, rno-miR-16-3p, rno-miR-495, rno-miR-6319
Naa15	4	rno-miR-202-5p, rno-miR-204-5p, rno-miR-21-3p, rno-miR-370-3p
Pcdhac1	4	rno-miR-127-5p, rno-miR-21-3p, rno-miR-495, rno-miR-743a-5p
Pcdha2	4	rno-miR-127-5p, rno-miR-21-3p, rno-miR-495, rno-miR-743a-5p
Pcdha12	4	rno-miR-127-5p, rno-miR-21-3p, rno-miR-495, rno-miR-743a-5p
Pcdhac2	4	rno-miR-127-5p, rno-miR-21-3p, rno-miR-495, rno-miR-743a-5p
Itpr1	4	rno-miR-127-5p, rno-miR-204-5p, rno-miR-21-3p, rno-miR-743b-5p
Pcdha10	4	rno-miR-127-5p, rno-miR-21-3p, rno-miR-495, rno-miR-743a-5p
Nr3c1	4	rno-miR-16-3p, rno-miR-200a-3p, rno-miR-204-5p, rno-miR-21-3p
Rapgef2	4	rno-miR-127-5p, rno-miR-16-3p, rno-miR-200a-3p, rno-miR-202-5p
Rhoq	4	rno-miR-134-5p, rno-miR-495, rno-miR-6319, rno-miR-743b-5p
Klhl24	4	rno-miR-134-5p, rno-miR-204-5p, rno-miR-370-3p, rno-miR-6319
Synj2bp	4	rno-miR-134-5p, rno-miR-204-5p, rno-miR-21-3p, rno-miR-495

**Note:**

miR, micro RNA.

### Results of DEMs expression collected by RT-qPCR

Real time quantitative polymerase chain reaction results supported the expression pattern of miRNAs obtained by high throughput sequencing, with the exception of rno-miR-204-5p, rno-miR-21-3p and rno-miR-6319. In all DEMs, rno-miR-370-3p was not detected by RT-qPCR as its expression was very low (Table 4).

**Table 4:** Relative quantifications of 16 differentially expressed miRNAs expressions detected by RT-qPCR.

DEMs	Group 2	Group 4	*p*-Value
rno-miR-370-3p	Not detected	Not detected	Not detected
rno-miR-200a-3p	1 ± 0.21	7.65 ± 2.33	0.038
rno-miR-127-5p	1 ± 0.19	20.27 ± 3.34	0.01
rno-miR-200b-3p	1 ± 0.20	6.77 ± 1.80	0.03
rno-miR-434-5p	1 ± 0.17	3.68 ± 1.04	0.044
rno-miR-743b-5p	1 ± 0.14	2.09 ± 0.32	0.005
rno-miR-341	1 ± 0.05	7.47 ± 1.15	0.01
rno-miR-495	1 ± 0.43	5.61 ± 0.69	0.001
rno-miR-337-5p	1 ± 0.06	3.55 ± 0.36	0.006
rno-miR-300-3p	1 ± 0.11	31.03 ± 0.75	0
rno-miR-6319	1 ± 0.16	1.40 ± 0.09	0.018
rno-miR-21-3p	1 ± 0.14	0.15 ± 0.02	0.001
rno-miR-16-3p	1 ± 0.13	2.49 ± 0.26	0.001
rno-miR-204-5p	1 ± 0.20	1.13 ± 0.07	0.347
rno-miR-743b-5p	1 ± 0.16	9.03 ± 1.59	0.012
rno-miR-134-5p	1 ± 0.16	20.12 ± 3.10	0

## Discussion

Acute increases in wall stress induced by pressure overload resulted in LV dilatation and led to VA. To our knowledge, we are the first to use this newly established and stable model to identify the antiarrhythmic effects and side effects of ART and VER. In this study, PVCs and VTs were detected promptly and no VFs occurred in response to LV afterload increases. Based on the results described previously, we concluded that an elevation in LV wall stress is more likely to induce VA in vivo. We detected that both ART and VER could effectively reduce the severities of life-threatening VA in response to LV afterload increases. Antiarrhythmic effects of ART on VA in response to LV afterload increases was found to be associated with the effects of ART on potassium and calcium ion channels as proved previously (Ai et al., 2001; Yang et al., 1998). AVBs were not associated with acute elevations in LV wall stress but emerged in the rats administered ART and VER. In ART- and VER-pretreated groups, type I second and two to one second degree AVBs were the most frequently observed AVBs.

At present, as the molecular mechanisms of artemisinin on preventing VA induced by afterload increases are unknown, DEMs-related molecular functions and signaling pathways may be an effective way to explore the underling mechanisms. Based on GO molecular functions, organ morphogenesis, dephosphorylation, neuron projection, axon, protein domain specific binding and transcriptional activator activity and RNA polymerase II transcription regulatory region sequence-specific binding were predicted to be associated with preventive effects of ART on VA in response to LV afterload increases. According to the results of KEGG pathways, the primary pathways may related to ART pretreatment were proteoglycans in cancer, the MAPK signaling pathway, the Wnt signaling pathway, lysosome and the Hippo signaling pathway, which could regulate arrhythmogenesis.

Stress-activated MAPK pathways, which comprise two subfamilies, the c-Jun NH2-terminal kinases (JNKs) and the p38-MAPKs, are sensors of pressure overload. Previous studies indicated that an abrupt increase in LV wall stress can activate a wide array of parallel pathways in vivo, especially JNK and p38-MAPK pathways. TAC results in hemodynamic stress, which produces an insignificant activation of ERK2 and subsequently, considerable increases in JNK1 and p38-MAPK events are detected (Fischer et al., 2001). Other studies validated reports that suggest abrupt pressure overload induces ERK, p38-MAPK and JNKs activations in the amphibian heart using the Langendorff model in vitro (Aggeli et al., 2001). In view of these research findings which were previously validated, we will further exploit the relationship between verified DEMs and DEMs related signaling pathways.

As was shown in predicted miRNA-mRNA network (Fig. 7), by integrating miRNA-related network, the current study provides a computational-based systems biology approach for further investigating the molecular mechanism of ART on VA in response to LV afterload increases. Genes such as Rere, Pcdha5, Ntrk2, Pcdha6 and Zfhx4 were targeted by multiple miRNAs (four and more miRNAs) as shown in Fig. 8.

## Study Limitation

No further research was executed to indicate the specific signaling pathways of DEMs related to the antiarrhythmic effects of ART on VA in response to LV afterload increases. In our future experiment, we will validate the relationship between DEMs and DEMs related signaling pathways and their effects on mechanical ventricular arrythmias.

## Conclusions

The findings of our study confirmed that both ART and VER can inhibit VA in response to LV afterload increases. The side-effects of ART and VER include type I second degree AVB and two to one second degree AVB. This research is a pilot study as our findings revealed the inhibitory effects of ART on VA in response to LV afterload increases for the first time.

These findings imply that dysregulated expressions of miRNAs and their potential target mRNAs are prominently involved in the antiarrhythmic effects of ART, suggesting that dysregulated miRNAs may serve as potential biomarkers and therapeutic targets of ART on mechanical VA. Predicting results of miRNA profiles could provide novel insights into roles of miRNAs related to preventive effects of ART on VA in response to LV afterload increases. However, further studies are needed to validate the relationships and mechanisms between miRNAs and their predicted signaling pathways.

## Supplemental Information

10.7717/peerj.6110/supp-1 Supplemental Information 1 16 differentially expressed miRNAs expressions validated by RT-qPCR.

10.7717/peerj.6110/supp-2 Supplemental Information 2 PR. Each data indicates PR intervals of every group.

10.7717/peerj.6110/supp-3 Supplemental Information 3 QTc. Each data indicates the QTc internal of every rat.

10.7717/peerj.6110/supp-4 Supplemental Information 4 The first column represents seven groups of rats named as group 1, 2, 3, 4, 5, 6 and 7, respectively. The second column represents the VAS of each rat. VAS: ventricular arrhythmia score.

## Additional Information and Declarations

### Data Availability

The following information was supplied regarding data availability: The raw data are provided in the Supplemental Dataset Files.
